# Characterization of Sensory-Motor Behavior Under Cognitive Load Using a New Statistical Platform for Studies of Embodied Cognition

**DOI:** 10.3389/fnhum.2018.00116

**Published:** 2018-04-06

**Authors:** Jihye Ryu, Elizabeth B. Torres

**Affiliations:** ^1^Sensory Motor Integration Laboratory, Department of Psychology, Rutgers University, Piscataway, NJ, United States; ^2^Computational Biomedical Imaging and Modeling Center, Department of Psychology and Computer Science, Rutgers University Center for Cognitive Science, Rutgers University, Piscataway, NJ, United States

**Keywords:** embodied cognition, cognitive load, heart rate variability, sensory-motor integration, pointing movements, stochastic processes

## Abstract

The field of enacted/embodied cognition has emerged as a contemporary attempt to connect the mind and body in the study of cognition. However, there has been a paucity of methods that enable a multi-layered approach tapping into different levels of functionality within the nervous systems (e.g., continuously capturing in tandem multi-modal biophysical signals in naturalistic settings). The present study introduces a new theoretical and statistical framework to characterize the influences of cognitive demands on biophysical rhythmic signals harnessed from deliberate, spontaneous and autonomic activities. In this study, nine participants performed a basic pointing task to communicate a decision while they were exposed to different levels of cognitive load. Within these decision-making contexts, we examined the moment-by-moment fluctuations in the peak amplitude and timing of the biophysical time series data (e.g., continuous waveforms extracted from hand kinematics and heart signals). These spike-trains data offered high statistical power for *personalized* empirical statistical estimation and were well-characterized by a Gamma process. Our approach enabled the identification of different empirically estimated families of probability distributions to facilitate inference regarding the continuous physiological phenomena underlying cognitively driven decision-making. We found that the same pointing task revealed shifts in the probability distribution functions (PDFs) of the hand kinematic signals under study and were accompanied by shifts in the signatures of the heart inter-beat-interval timings. Within the time scale of an experimental session, marked changes in skewness and dispersion of the distributions were tracked on the Gamma parameter plane with 95% confidence. The results suggest that traditional theoretical assumptions of stationarity and normality in biophysical data from the nervous systems are incongruent with the true statistical nature of empirical data. This work offers a unifying platform for personalized statistical inference that goes far beyond those used in conventional studies, often assuming a “*one size fits all model*” on data drawn from discrete events such as mouse clicks, and observations that leave out continuously co-occurring spontaneous activity taking place largely beneath awareness.

## Introduction

Cognitive Science as a field has focused primarily on the study of the mind, with few studies addressing the mind-body interactions. In recent years, the field of embodied cognition has emerged to fill this gap and try to connect mental representations with physically enacted actions (Wilson, 2002; Mahon and Caramazza, 2008). However, progress in this nascent field has stalled, partly because there are no proper ways to statistically quantify cognition and action under a common framework. Given that motor action is a result of the central and peripheral nervous systems working together, it is necessary to study continuous output signals from all layers of the nervous systems in tandem. These include the brain, the heart and the body in motion. Conventional studies are often based on *discrete* epochs of biophysical signals obtained during constrained (unnatural) actions whereby decisions are marked by mouse clicks, self-reports and/or observation. Such classes of actions contrast with signals obtained during naturalistic and self-generated *continuous* behaviors.

**Figure 1:**
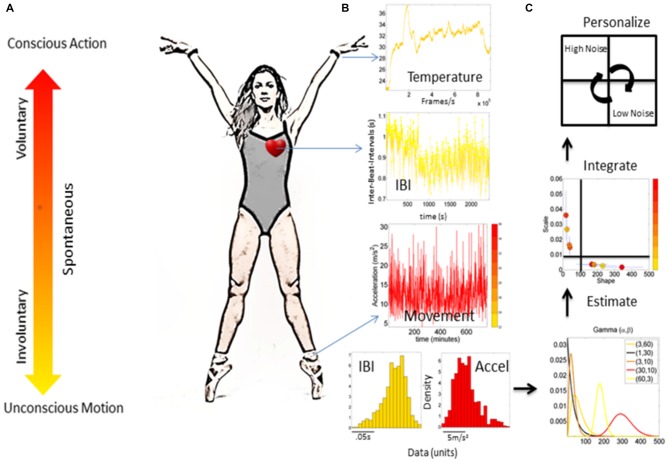
Micro-movements from different nervous systems’ biorhythms. **(A)** Continuum of different levels of control ranging from those that are conscious and goal-directed to those that are autonomic and unconscious (taken from Torres, 2011). **(B)** Different waveforms from different instrumentations (e.g., heart activity, temperature and movement) registered from physiological sensors. Raw biophysical signals give rise to a time series of peaks and valleys, which vary in amplitude and timing. The fluctuations in the amplitude and timing of the peaks are the “micro-movements” of biophysical signals. **(C)** The micro-movements datatype can be used in an empirical estimation of families of probability distributions (e.g., using a Gamma process) to estimate parameters of the probability distribution functions (PDFs), track the integrated signatures from different layers of the nervous systems on the Gamma parameter plane, and separate regimes of low vs. high noise-to-signal ratio (NSR), and low vs. high symmetry of the distribution based on the shape values.

In the naturalistic case, spontaneous movement segments coexist with deliberate ones and are performed largely beneath the person’s awareness (Torres, 2011). Indeed, naturalistic actions involve varying levels of functional control, which range from those that are intentional and goal-directed, to those that are autonomic in nature (Figure 1A). Activities of daily living require the coordination and control of motions along all these levels of functionality. Accordingly, it is important to understand the evolving dynamics of biophysical signals across the multiple layers of the nervous systems, under different levels of functional control within these systems. To that end, the current study introduces a new theoretical and methodological framework that assesses the influences of cognitive loads on bodily motions. We use the hand’s and the heart’s rhythmic motions during continuously repeated pointing gestures to indicate cognitive-loaded decisions.

In order to understand the interactive dynamics across the different nervous systems, we introduce a new theoretical framework (Torres et al., 2013a) grounded on the principle of reafference (Von Holst and Mittelstaedt, 1950), from the works of Von Holst and Mittelstaedt, stating that “*Voluntary movements show themselves to be dependent on the returning stream of afference which they themselves cause*.” Our work expands the use of this principle to other non-voluntary movements’ functionalities and to movements that may be independent of the returning afferent stream but coexisting within voluntary actions. These include supportive motions that occur spontaneously and do not pursue a goal, involuntary motions inherent in the person’s system, automated and autonomic motions. These motions have different dynamics and funnel differently the influences of dynamics on the geometry of the paths their trajectories describe—as compared to those intended to a goal, i.e., deliberately performed with intent or purpose (Torres, 2001, 2010, 2011; Torres and Zipser, 2004; Torres and Andersen, 2006; Torres et al., 2013c). They also have the common feature that the person is less aware of them than those performed under voluntary control.

Within this framework, studying physiological signals with varying levels of functional control, it is then essential to understand co-existing levels of functionality permeating the closed feedback loops between the CNS, the PNS, and within the PNS, the ANS. These multi-modal flows of information exert influences over one another. For instance, it has been shown that spontaneous actions (e.g., retracting motion of the pointing hand) co-exist with, and are instrumental to the goal-directed segments of complex motions, as they provide fluidity to behavior at large (Torres, 2011). Along those lines, prior work concerning neuromotor features of complex actions with coexisting multi-functional movement segments examined the interplay between deliberate and spontaneous movements to characterize their stochastic signatures among athletes vs. novices (Torres, 2011, 2013b). Within the realm of basic perceptual science, the new framework has been used to examine top down influences of visual illusions on multi-functional motor control (Nguyen et al., 2013, 2014a,b). In the health space, these new methods under the aforementioned theoretical construct have been used to examine individuals with autism spectrum disorders (Torres, 2013c; Torres et al., 2013a), schizophrenia (Nguyen et al., 2016), Parkinson’s disease (Torres et al., 2011), stroke (Torres et al., 2010) and deafferentation (Torres et al., 2014). More generally, these methods have been deployed as a new platform for personalized medicine drawing on principles of the Precision Medicine approach (Torres et al., 2016a,b) for Big Data analyses (Torres and Denisova, 2016; Torres et al., 2017) and mobile health concepts (Torres, 2013a; Torres and Lande, 2015; Torres et al., 2016c). The use of the fluctuations in amplitude and timing extracted from parameters in biophysical signals provides a proper level of detail to detect preferences in sensory guidance and help evoke and steer the system’s autonomy, its volitional control and ultimately its agency (Figures 1B,C).

We posit that interactions among signals from the full range of functionalities and from different nervous systems are necessary for the development and maintenance of *deliberate autonomy* (i.e., the ability to deliberately maintain a robust course of action on demand, impervious to external/environmental influences).

Under the lens of this framework, to examine how increase in cognitive demands are manifested across different nervous systems, we assessed the variability inherent in the biophysical rhythms that we harnessed noninvasively from the various layers of the nervous systems. We characterize the influences of increases in cognitive demands on the hand movement kinematics and the heart signals, using new statistical methods under the renovated kinesthetic reafference framework, as applied to the multi-layered and multi-functional nervous systems. Here, we vary the level of cognitive demands during a pointing task to communicate a decision. Under those conditions, we examine: (1) the goal-directed segment of the pointing motion; (2) the supplemental segments of the retracting motions; and (3) the heart rate variability, as these provide a window into the individual’s mental states during cognitive decisions, in an interactive closed-loop between the central and the peripheral nervous systems.

## Materials and Methods

### Participants

Nine undergraduate students (two males and seven females) between the ages 18 and 22 were recruited from the Rutgers human subject pool system and received credit for their participation. This study took place at the Sensory Motor Integration Laboratory of Rutgers University. All participants signed the consent form approved by the Rutgers University Institutional Review Board (IRB). The entire study protocol was approved by the Rutgers University IRB. The study conforms to the guidelines of the Helsinki Act for the use of human subjects in research. Two participants were left-handed, and all had normal or corrected-to-normal vision.

During the experiment, the motor and heart signals were recorded from each participant. However, one participant’s heart signals did not record successfully due to instrumentation malfunctioning, resulting in an analysis on motor data for nine individuals and heart data for eight individuals.

### Sensor Devices

In this study, two sensor devices—motion capture system and a wireless heart rate monitor—were used to record the signals coming from the bodily movements and the heart. The data obtained from these two devices were analyzed separately.

#### Motion Capture

Fifteen electromagnetic sensors at a sampling frequency of 240 Hz (Polhemus Liberty, Colchester, VT, USA) were used to continuously capture the participant’s movements across the upper body. Nine sensors were placed on the following body segments using sports bands to optimize unrestricted movement of the body: center of the forehead, thoracic vertebrate T7, right and left scapula, right and left upper arm, right and left forearm, the dominant hand’s index finger. An additional sensor was used to digitize the body in constructing a biomechanical model using the Motion Monitor (Innovative Sports Training Inc., Chicago, IL, USA) software. One sensor was placed at the backside center of the iPad (Apple, Cupertino, CA, USA) display screen. This sensor served to measure the physical position of the fixed target, to help obtain a distance-based criterion to automatically classify motions into forward (from the resting position of the hand to the target) and backward (from the target to the resting position). There were also four positional sensors placed at the four corners of the table on which the iPad was standing. This physical information enables us to build computational models of these movements to study Bernstein’s degree of freedom problem (Torres and Zipser, 2002; but that work is beyond the scope of this article). During the experiment, the participant’s motion was captured in real-time, recording the location and speed of the upper body movements.

#### Heart Rate Monitor

Heart signals were obtained via electrocardiogram (ECG) from a wireless Nexus-10 device (Mind Media BV, Netherlands) and Nexus 10 software Biotrace (Version 2015B) at a sampling rate of 256 Hz. Three electrodes were placed on the chest according to the standardized lead II method and were attached with adhesive tape. A typical ECG data includes a set of QRS complexes and detecting R-peaks (within the QRS complex) is essential, as the heart rate metrics needed for this study focuses on the oscillation of intervals between consecutive heartbeats. To remove any baseline wandering and to accurately detect the R-peaks, ECG data were preprocessed using the Butterworth IIR band pass filter for 5–30 Hz at 2nd order. The range of the band pass filter was selected based on the finding that a QRS complex is present in the frequency range of 5–30 Hz (Kathirvel et al., 2011). To retrieve the time between R-peaks (i.e., inter-beat intervals, IBI) from the preprocessed ECG data, simple peak detection method was used, and was plotted using Matlab graphics to ensure that there were no missed R-peaks.

### Stimulus Apparatus and Experimental Procedure

Once all sensors were donned and calibrated, participants were seated at a table facing an iPad used as a touchscreen display. An in-house developed MATLAB (Release 2015b, The MathWorks, Inc., Natick, MA, USA) program controlled the presentation displayed on the touchscreen display and recorded the timing and location of the touches made by the participant. The MATLAB program was presented on the touchscreen display using the TeamViewer (Germany) application.

**Figure 2:**
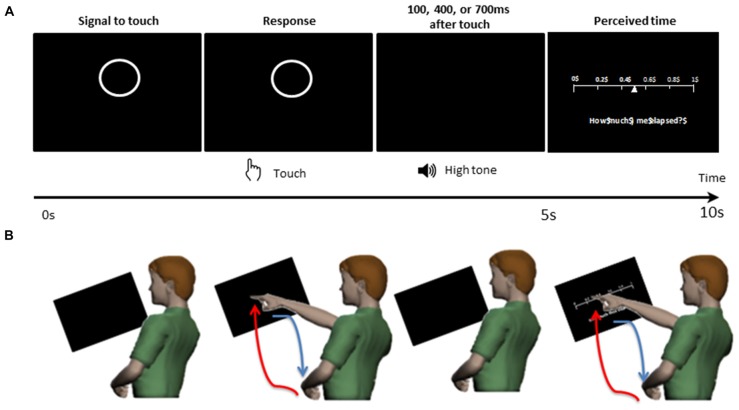
Experimental design. The participant was presented with a display screen as shown on **(A)**. During the first 5 s, the screen presented a circle prompting the participant to touch the circle on the screen. After the touch, the participant heard a tone. The duration between the touch and the tone was randomly set to be 100 ms, 400 ms, or 700 ms. In the next 5 s, the participant was presented with a sliding scale, where the participant would indicate how long they perceived the time to have elapsed between the touch and the tone, by touching the corresponding number on the scale. **(B)** For each trial, the participant made a pointing gesture to touch the circle and to indicate their time estimation on the sliding scale. The pointing movement is composed of a voluntary/goal-directed forward segment (red) and a spontaneous backward segment (blue).

As shown in the schematics of Figure 2, for each trial, the participant was presented with a circle on the center of the display screen. This presentation prompted the participant to touch the circle on the screen within 5 s. After the touch, the participant heard a tone at 1000 Hz for 100 ms. The duration between the touch and the tone was randomly set to be 100 ms, 400 ms, or 700 ms. Then, on the display screen, the participant was presented with a sliding scale ranging from 0 to 1. On the sliding scale, the participant indicated how long they perceived the time to have elapsed between the touch and the tone, by touching the corresponding number on the scale within 5 s. Note, the 5 s time-windows allowed ample time for the participant to touch the screen at their own pace, as the time to reach the screen and then to retract the hand took approximately 1.5 s. Supplementary Table S1 of the Supplementary Material summarizes the median time to move the hand under each condition.

The experiment consisted of three conditions—control, low cognitive load and high cognitive load condition. Under the control condition, the participant simply performed this task for 60 trials. Under the low cognitive load condition, the participant performed these tasks for 60 trials, while repeatedly counting out loud one through five. Under the high cognitive load condition, the participant performed these tasks for 60 trials, while counting backwards from 400 subtracting by 3.

Participants performed the conditions in the order of control-baseline, low cognitive load condition, and high cognitive load condition. Note, the order was not counterbalanced, because performing high cognitive load tasks prior to low load tasks would have caused cognitive load and fatigue to be carried over to the low cognitive load condition. This might have influenced the effect of cognitive load to be mixed with fatigue, but this was necessary to keep the low cognitive load tasks to be minimally taxing as possible. For both low and high cognitive load conditions, the participant was instructed to count at a comfortable pace. Participants took breaks in between conditions, and the entire experiment took about 40 min.

### Justification and Assessment of Levels of Cognitive Load

**Figure 3:**
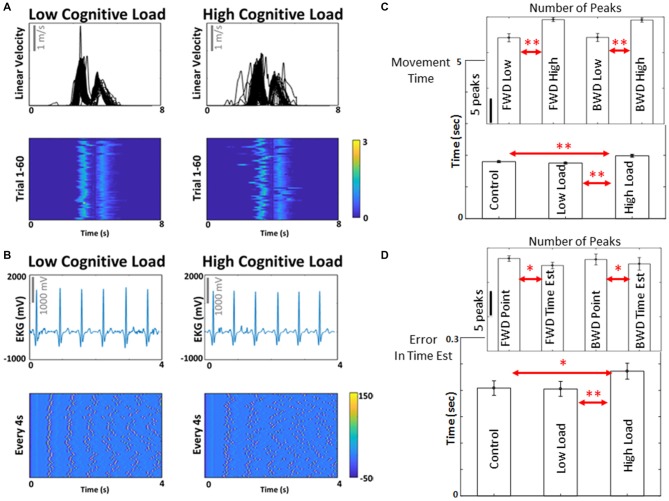
Biorhythms from different nervous systems and motor/behavioral results from cognitive load. **(A)** Biorhythm of motor signals, from hand pointing movements, in the form of temporal speed profiles across 60 trials, exhibit moment by moment variations with different levels of cognitive load. Motions are aligned to the touch of the screen and heat maps are used to show the speed peaks (cm/s) for the forward and backwards motions. Peaks of the electro-cardiogram signals (ECG) are aligned (4 s) and represented in **(B)** as spikes. Later in the analyses, these peaks become standardized as unit-less micro-movements ranging on the real-valued scale from 0 to 1 for further stochastic analyses (see “Materials and Methods” section). **(C,D)** To validate the effect of cognitive load, movement time (i.e., time was registered from the time when the participant was prompted to reach the target, to time of completion of the reach by touching the screen), error in time estimation, and average number of angular acceleration peaks per trial were compared between the high and low cognitive load conditions, and between pointing and time estimation tasks. Movement time showed significant difference between control and high cognitive load condition (*t*_(8)_ = 3.53, *p* < 0.01) and between low and high cognitive load condition (*t*_(8)_ = 0.15, *p* < 0.01). Error in time estimation also showed significant difference between control and high cognitive load condition (*t*_(8)_ = 2.89, *p* = 0.04) and low and high cognitive load condition (*t*_(8)_ = 4.21, *p* < 0.01). Number of angular acceleration peaks were significantly different between low and high cognitive load conditions for forward motions (*t*_(8)_ = 5.4, *p* < 0.01) and backward motions (*t*_(8)_ = 7.6, *p* < 0.01); and between pointing and time estimation tasks for forward motions (*t*_(8)_ = 2.2, *p* = 0.05) and borderline significant for backward motions (*t*_(8)_ = 2.1, *p* = 0.07). **p < 0.01; *p < 0.05. The experimental paradigm described in Figure 2 proved efficient to probe cognitive demands and characterize cognitive loads by time series of peaks in trajectories described by the hand’s angular acceleration. See Supplementary Tables S2–S4 for all pairwise comparisons of these metrics.

To illustrate the effects of subtle increases in cognitive demands on the hand kinematics, we use Figure 3A where the hand speed profiles for the low and high cognitive load conditions show marked differences in variability as the movements unfold in each trial, and as they are performed from trial to trial. Besides the speed profiles, these differences can be appreciated in plots of heat maps where the peaks are highlighted for 60 trials. Indeed, the results extend to the heart beat, as illustrated in Figure 3B using similar format as in Figure 3A (i.e., the waveform of the raw signal and the heat map of the peaks).

To justify the use of this paradigm to test influences of cognitive demands on movement kinematics, we evaluated the number of peaks in the angular acceleration waveform for both the deliberate portion of the reach (forward to the target) and the spontaneous retraction (backward to rest).

The effects of the increase in cognitive demands manifested in statistically significant changes in the accuracy of the task and the time of performance. The group incurred a significant increase in the time to point (*t*_(8)_ = 0.15, *p* < 0.01) explained by the statistically significant increase in the number of angular acceleration peaks as the cognitive demands increased. The accumulation of peaks in the rates of hand angular speed with higher cognitive load resulted in higher physical effort, as the participants had longer angular excursions with higher cognitive demands. The insets in Figure 3C show the increase in number of peaks for both types of motions under consideration (forward and backwards). Lastly, we confirmed that the increase in cognitive demands affected their accuracy in estimating time. This is shown in Figure 3D, where the increase in the cognitive loads resulted in statistically significant higher errors of the time estimation (*t*_(8)_ = 4.21, *p* < 0.01). All these preliminary tests confirmed that the motor task we designed was adequate to assess variations in cognitive load and their potential effects on physiological parameters of interest. We then proceeded to examine these physiological waveforms’ peaks in terms of spike trains under the general rubric of continuous stochastic processes.

### Data Analysis

#### The Statistical Platform for Individualized Behavioral Analyses (SPIBA)

The current study employs a new platform, Statistical Platform for Individualized Behavioral Analyses (SPIBA; Torres and Jose, 2012), which was created for personalized assessments required in the Precision Medicine and mobile Health concepts (Hawgood et al., 2015). For the present study, the SPIBA was used to first characterize each participant individually, which could potentially be used to automatically (without heuristics of e.g., machine learning algorithms to classify labeled data) identify self-emerging clusters of participants based on their similar statistical patterns in subsequent studies. This platform stands in stark contrast to current approaches in health sciences (e.g., significant hypothesis testing method), which tend to compare hand-picked grouped data under some inclusion/exclusion criteria and assumed to follow a normal distribution with homogenous variance. The pitfalls of such methods have been discussed by others (Gallistel, 2009; Gallistel and King, 2009) and the Bayesian framework has been offered (e.g., in fields of Cognitive Science and Neuroscience) as an alternative to address some of the known weaknesses of traditional approaches to statistical inference. However, the Bayesian approach has not been adapted to analyze multiple types of biophysical data in tandem, obtained from different layers of the nervous systems, including those that are internally generated with disparate levels of functionality.

The SPIBA framework, with the use of a new datatype coined “*the micro-movements*” of biophysical signals (explained in the following section), was precisely designed to longitudinally tackle the emergence, dynamic development, maintenance and degeneration of the signals produced by the multi-layered nervous systems, including those with different pathologies over the human lifespan (Torres et al., 2016a).

#### New Data Type: Definition of Micro-Movements

The raw biophysical data continuously registered from physiological sensors (i.e., physiological signals obtained by ECG, respiration patterns, kinematics from bodily, head and eye movements, tremor data, etc.) give rise to time series of peaks and valleys, which vary in amplitude and timing (Figure 3). The fluctuations in amplitude and timing of the peaks are treated as spikes and assumed to follow a continuous random process where events in the past may (or may not) accumulate evidence towards the prediction of future events. Under this framework, we distinguish the processes, whereby the consequences of the signals *dependent on the returning stream of afference which the (voluntary) motions themselves cause*, from the independent processes. The latter are those for which the present events are independent of the past events. All fluctuations treated as standardized spikes in the 0–1 unit-less real number range are the “*the micro-movements*” of biophysical signals. To model them, we build on our original work (Torres et al., 2013a) whereby random variables follow a Gamma process (rationale behind this is explained in next).

For the current study, the goal is to show an example of using SPIBA and micro-movement data involving signals harnessed in tandem from the PNS and ANS while performing CNS driven decisions. To that end, we will examine biophysical data from body movements and the heart activities in a personalized fashion, as each participant is exposed to a decision-making task with different levels of cognitive load.

#### Different Classes of Movement Segments—Forward vs. Backward

**Figure 4:**
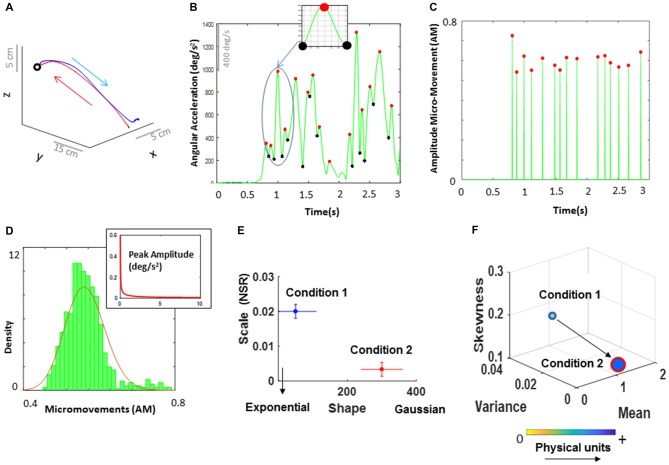
Analytical and visualization methods. **(A)** Continuous positional trajectory of the dominant hand performing a single pointing movement loop forward to the target (instructed) and backwards to rest (spontaneous). Forward motion corresponds to the movement from the time when the index finger is resting on the table and lifts to move until the time the finger touches the target displayed on the screen and stops. The backward movement corresponds to the movement from the time the index finger leaves the target and retracts back to the table. **(B)** Time series of angular acceleration of the dominant hand’s index finger rotations during a typical pointing task. Peaks (maxima) and valleys (minima) are shown in red and black dots, respectively. The inset shows a zoomed-in picture of a single angular acceleration segment (i.e., two local minima and a single local peak in between). This is a schematic of computing the amplitude micro-movements (AM; i.e., normalized peak amplitude) from a continuous time series of signal data, where the AM is computed by dividing the peak value by the sum of the peak value and the average of the signal values between the two local minima (see equation 1). **(C)** Spike train for a typical pointing task. All peak values from **(B)** are normalized between 0 and 1, while all non-peak values are set to 0. **(D)** All AM values were identified and gathered across all trials. For these data, a frequency histogram was then plotted, and fitted with a Gamma PDF using maximum likelihood estimation (MLE). In addition to the AM values (used here to explain these methods), we can also plot an inset PDF of the raw data (i.e., angular acceleration or time to peak TM) to show the differences in PDF between the raw data and the data scaled by equation (1). **(E)** The estimated Gamma parameters from the fitted probability distribution corresponding to the parameters registered in two sample conditions (e.g., high load and low load) were then plotted on a Gamma parameter plane, with lines representing the 95% confidence interval (CI). **(F)** Empirically estimated Gamma moments: mean, variance, and skewness plotted on the x, y, and z axes respectively. The size of the marker reflects the level of kurtosis, where larger size indicates high kurtosis level of the fitted PDF. The arrows connecting the markers indicate the order of the task conditions. The marker’s face color represents the median values of the underlying physical units (e.g., the range values of deg/s^2^). For example, the marker with blue edge representing Condition 1, is yellow, signaling a color of lower values in the color bar than the blue color of the marker with red edge representing Condition 2. This representation to visualize the data means that from Condition 1 to Condition 2, the skewness dropped towards the 0-value reference for symmetric distributions (also marked by a right-shift in **(E)** along the shape axis); a decrease in kurtosis (peakier distribution in Condition 2 than in Condition 1) with a drop in the NSR in **(E)** along the scale axis from Condition 1 to Condition 2. Further, the shift from Condition 1 to Condition 2 shows an increase in the mean with a reduction in the variance (explaining the drop in NSR, i.e., mean/var).

The continuous positional trajectory of the participant’s dominant hand index finger was decomposed into forward and backward movements (schematics of Figure 2 (bottom panel) and sample hand movement trajectory in Figure 4A). The forward movement corresponds to the movement when the hand resting on the table would reach out to touch the display screen. As this movement involves an explicit goal in mind (i.e., to touch the display screen), this movement involves a high level of intention. On the other hand, the backward movement corresponds to the movement when the hand touching the display screen would spontaneously (without any instruction) retract back to the table. Because this uninstructed movement does not involve an explicit goal and is more automatic, it involves a relatively lower level of intention. Note, this forward-retraction motion paradigm has been developed (Nguyen et al., 2014a) and translated in clinical (Torres et al., 2010, 2011, 2013a,b, 2016a; Yanovich et al., 2013; Hong et al., 2014; Amano et al., 2015; Nguyen et al., 2016) and sports research (Torres, 2011).

For each trial, as the participant moved the dominant hand from the table to the display screen and back to the table, the movement trajectory consisted of a single forward and backward movement segment. Within the trajectory, the two movement segments were extracted, by identifying the time when the distance between the index finger and the display screen was at the minimum. Naturally, the linear velocity of the index finger reaches near instantaneous zero at that point. Hence, the forward movement would correspond to the movement from the time when the index finger is resting on the table until the time the finger stops at the display screen. The backward movement, on the other hand, would correspond to the movement from the time when the index finger stops at the display screen until it reaches back to the table and rests (i.e., the speed value is near zero again).

As explained above, the rationale behind the separation between forward and backward movement is that one is instructed and goal-directed, while the other is not, thus differing in their levels of intent. The latter is spontaneously self-initiated by the person without instruction. The statistical characteristics have been shown to differ between forward and backward movements (i.e., motion segment with high vs. low level of intent) during reaching, pointing, and grasping actions among different patient populations and across the general human population (Torres et al., 2010, 2011, 2013a, 2014; Nguyen et al., 2016). For that reason, we expect that separating the movements in such a manner would allow us to examine the impact of cognitive load on movements involving different levels of intent.

Analyses of the sensors from other body parts are beyond the scope of this article and will be disseminated in future work.

#### Motivation and Rationale: Micro-Movements Analytics for Motor Signals

For each forward and backward movement, we examined the linear and angular positional data and their higher order derivatives: the linear velocity, the angular velocity, the linear acceleration and the angular acceleration. For each time-series data the peak amplitudes and inter-peak intervals were identified, converted to micro-movements (see below) and gathered across all trials. Among the four types of parameters, for both forward and backward movements, angular acceleration was analyzed, as it has the largest number of peaks and provides the signal with the highest statistical power (hundreds of peaks per person) to carry on our stochastic estimation with high (95%) confidence.

We underscore that the current paradigm relies on the statistical power of an estimation procedure (which will be detailed in the next paragraph) so the higher the number of samples used to make an empirical estimation for a given person, the less taxing the experiment is to the participant, as it takes less time to attain a robust estimate. For instance, during a typical point-to-point reaching action, which consists of a single forward and backward movement, the linear velocity would typically provide at least two salient samples (peaks; see Figure 3A), one for forward and one for backward movement. To gain enough peak data from the linear velocity speed profile during a single experimental session and attain proper statistical power, the participant would need to perform at least 100 reaches. These would give us statistical power for the estimation of the probability distribution function (PDF) describing each segment but would likely lead to fatigue-related effects. However, using underlying kinematic parameters with higher number of samples (i.e., higher order of peak data) instead can result in shorter experiments. In turn, this would allow us to include additional conditions to manipulate various contextual parameters. For that reason, the current study focused on examining the peak data obtained from *angular acceleration*, as this provides the most power in the statistical estimation within the shortest time. The tradeoff here is that higher order derivatives of the position/orientation data (such as angular acceleration) can introduce large fluctuations from instrumentation noise. However, we have developed in house filtering/smoothing methods (Nguyen et al., 2014a) and combined them with traditional filtering (Paarmann, 2001) to eliminate such potential issues when using higher order derivatives of the position and orientation data. In the present work, we further rely on a variety of filtering algorithms embedded in the data collection interface we used (Sports Inn, The Motion Monitor, Chicago, IL, USA).

To build a unit-less normalized scale, and to address possible allometric effects (Mosimann, 1970) due to individual anatomical differences, the peak amplitudes of the angular acceleration were normalized as:

(1) $$Norm\;Peak\;Amplitude\;(AM)=\frac{Peak\;Amplitude}{Peak\;Amplitude+Avrg_{\text{Min to Min}}}$$

Normalized peak amplitude (coined *amplitude micro-movements* (AM), Torres et al., 2013a) provides a scaled summary of the continuous data, and is computed using equation (1), by dividing each local peak amplitude by the sum of the peak amplitude and the average of the signals sampled within the neighboring points of two local minima surrounding the peak (Figure 4B inset). While we convert the analog continuous signal into a point process and treat it as a continuous random process for statistical estimation, the averaging in the denominator preserves the information contained in the points surrounding the maxima. Higher values of the AM imply lower values of the signal amplitude on average. Likewise, shifts towards lower values of AM imply increases on the magnitude of the amplitude values on average. We emphasize that representation of spike trains is not reduced to a binary scale (unlike the binary representation of cortical neuronal spikes). We work with a continuous (normalized) scale with real values ranging from 0 to 1. Further the averaged peaks in the denominator are Gamma distributed and so are the peaks in the numerator. As such the resulting scaled value is also Gamma distributed.

Besides the amplitude information, peak data can provide estimates related to the motion’s temporal dynamics. To that end, normalized inter-peak interval timings were computed by extracting the time elapsed between consecutive peaks (*timing* TM) and normalizing the array of these TM values using Equation 1 (coined *timing micro-movements* NTM). The two types of normalized, unitless spike-dependent data (i.e., AM, NTM) can be visualized in a spike train format as shown in Figure 4C. Further, the physical ranges (deg/s^2^ and seconds) of the original peaks can be used to color code the graphs and show the range of parameters of each participant as shown in Figure 4F. This is a personalized approach that enables us to distinguish the physiological features of each person, while automatically unveiling self-emerging trends in a group.

The micro-movements are then used as input to a Gamma process. These spike-train data are accumulated within a time window that depends on the sampling resolution of the sensors and on the physical phenomena under investigation. In this case, the sampling resolution is 240 Hz and the physical phenomena (i.e., a self-generated pointing movement consisting of a forward and backward motion, produced by the nervous system) are on the order of approximately 1500 ms each (see Supplementary Table S1). As such, we have many sample peaks within a single minute. Usually, we set the size of the sampling window to 1 min (e.g., when we collect data continuously for 12 h in a hospital setting; Torres and Lande, 2015), but in the present article we use all trials in a given condition accrued across the experimental session. The time window for the estimated statistical parameters is the duration of the session (see experimental epochs for one trial in Figure 2). For each condition we gather all peaks of the angular acceleration and plot a frequency histogram using optimal binning (Freedman and Diaconis, 1981; Shimazaki and Shinomoto, 2007; Figure 4D). The histogram is then fitted using maximum likelihood estimation (MLE; see Supplementary Figure S3) to estimate the best continuous family of probability distributions that fits the data with high confidence. We have set the confidence intervals (CIs) for the empirically estimated Gamma parameters to 95%.

Prior work from our lab was the first to explore in human data the differences between multiplicative (e.g., lognormal family) and additive (e.g., exponential families) random processes of the micro-movement spike trains during voluntary, automatic and involuntary motions (Torres, 2011). Among these are micro-movements data from boxing routines involving voluntary and spontaneously performed movements (Torres, 2011, 2013c), forward-retracting motor loops during target-directed reaches (Torres et al., 2010, 2011, 2013a, 2014; Nguyen et al., 2016), natural walking involving automatic gait patterns (Torres et al., 2016b), and involuntary head motions during resting state within fMRI experiments (Torres and Denisova, 2016; Torres et al., 2017). In all cases, the continuous Gamma family of probability distributions has been the best fit (based on MLE and Kolmogorov-Smirnov tests (KSTs) for empirically derived cumulative distributions), showing that the human data has a wide range of PDFs, ranging from the exponential to the normal distribution. This contrasts with the assumption of a one size fits all model guided by the theoretical Gaussian distribution. Given that we found good fitting for the Gamma family under MLE, here we opted for the Gamma process to represent our spike trains of micro-movements. The Gamma PDF is given by:

(2) $$y=f\left(x|a,b\right)=\frac{1}{\Gamma (a)b^{a}}x^{a\;-\;1}e^{\frac{-x}{b}}$$

in which *a* is the shape parameter, *b* is the scale parameter, and Γ is the Gamma function (Ross, 1996). The two parameters in equation (2)—shape (*a*) and scale (*b*)—were estimated for each histogram of the micro-movement data, as mentioned, using MLE with 95% CIs. The estimated parameters with their CI were plotted on a Gamma parameter plane, where the *x*-axis represents the shape parameter value and the *y*-axis represents the scale parameter value (Figure 4E).

The Gamma scale value conveys the noise to signal ratio (NSR) since the Gamma mean *μ*_Γ_ = *a*·*b* and the Gamma variance is *σ*_Γ_ = *a*·*b*^2^, the scale:

(3) b=NSR=σΓμΓ=a⋅b2a⋅b

In this sense, according to equation (3), the scale axis of the Gamma parameter plane allows us to infer behaviors leading to higher noise levels vs. lower noise levels. Along the shape axis, the Exponential distributions at *a* = 1 are found in autism cases (Torres, 2011, 2013c). Using this approach, we can track processes whereby events in the past do not contribute to the prediction of future events and are well characterized by the Exponential (the most random) distribution. We can also track processes where the events in the past predict future events with high certainty and observe skewed to symmetric distributions along the shape axis with the Gaussian distribution at the opposite extreme of the Exponential case. We have indeed done so and provided the first empirical characterization of human motions on the Gamma parameter plane (Torres et al., 2016a).

Additionally, the estimated Gamma moments were obtained and plotted in a four-dimensional graph (Figure 4F). Here, the empirically estimated mean, variance and skewness of the fitted Gamma PDFs are plotted on the x, y and z axes respectively. The size of the marker reflects the level of kurtosis, where larger size indicates higher kurtosis level (distributions with sharper peaks) of the fitted PDF. Negative skewness means that the data are spread out more to the left of the mean than to the right. Positive skewness means that the data are spread out more to the right. Zero skewness indicates a perfectly symmetric distribution. This four-dimensional graph allows us to visualize the statistical features of the micro-movements and understand how the stochastic signatures shift across different conditions and/or individuals. The arrows are included to indicate the orderly flow of changes across different conditions. Note, that we standardized the waveform to a unit-less real-number ranging from 0 to 1 and lost the original range of the physical units. To capture the physical range of the raw data for each person, we include color as a fifth dimension to visualize the gradient of physical ranges of the data underlying the AM (expressed in deg/s^2^) and NTM (expressed in second), giving us the change in physical units along this color gradient for each participant. The marker’s face represents the median of the physical values within a condition, and the marker’s edge is used as another feature to represent the condition (i.e., cognitive load type). This visualization tool allows us to see the physical ranges of each individual person in transition from one condition to another, while expressing all parameters along a common unit-less standard scale. Note that reporting on the physical parameter ranges of each instrument while maintaining the standardized unit-less scale for statistical estimation and inference is amenable for data exchange and reproducibility of results in our fields of study.

Graphs such as Figures 4E,F produce a useful visualization tool to uncover patterns (see Torres et al., 2016a, 2017) for examples of large population groups that self-cluster according to nervous systems pathologies using these methods). Here, we can visualize participants as a group and uncover self-emerging clusters of the general population, without* a priori* hand-picking homogeneous groups for significant hypothesis testing comparison (as it is traditionally done across the fields of brain and health sciences). This is very important because the patterns that we uncover are entirely *data-driven*, as the patterns self-emerge from the inherent variability of the nervous systems signals.

In the present work, we only have nine participants. However, we underscore the personalized and empirically driven nature of this statistical platform, as the statistical power lies in the number of samples per person. The population family of Gamma distributions for the human spectrum has been *empirically* characterized in prior work (Torres et al., 2016a) for this basic pointing task. This platform enables us to make well-informed statistical inferences and interpret the empirically-driven statistical phenomena under consideration.

Note also that we can examine the deliberate and spontaneous processes by analyzing the micro-movement of forward and backward motor signals, since the empirical data showed that these processes map well onto voluntary goal-directed motions and automatic uninstructed/goal-less motions, respectively (Torres, 2011, 2013c).

#### Analytics for Heart Signals (Inter-Beat Interval)

Like the analysis performed on the micro-movement peak data of motor signals (i.e., AM, NTM), we applied the distributional analyses on the IBI data for each condition. As with the hand kinematics, we fitted the PDF using MLE (see Supplementary Figure S4). Histograms for the IBI data were fitted among the Gamma, exponential, lognormal, and normal for each condition, and we determined that the continuous Gamma family of distributions would be appropriate for fitting the IBI data. For that reason, the parameters of the Gamma PDF were estimated for each histogram of the IBI data, and the shape and scale values were plotted on the Gamma parameter plane with 95% CIs, and the Gamma moments of the estimated PDFs were plotted on a 4D graph. Through this analysis, we could examine the inevitable processes emerging from the ANS.

## Results

Given the preliminary analyses of Figure 3C, demonstrating statistically significant effects given by increases in cognitive demands on the participants’ performance (accuracy and time), and their influences on the number of peaks of the biophysical signals, we felt confident to explore the stochastic nature of signals and more precisely characterize such effects at different levels of functionality and control.

### ANS Assessment of (Inevitable) Autonomic Control of IBI

**Figure 5:**
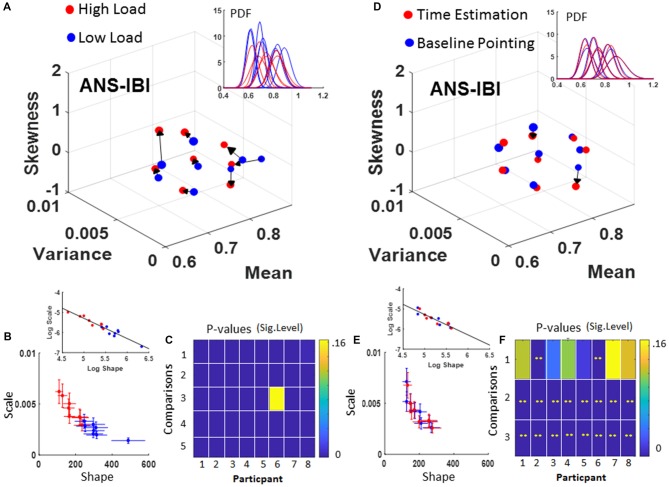
ANS autonomic control assessment under high and low cognitive load conditions. Inter-beat intervals (IBI) signal. When comparing between high and low cognitive load conditions **(A–C)** and basic pointing with pointing during time estimation **(D–F)**. **(A)** Shifts in the empirically estimated Gamma moments of the IBI distinguish Condition 1 (low load) from Condition 2 (high load) along moment axes, and PDFs spanning a family (inset). **(B)** Gamma parameters separate conditions whereby high load have higher NSR and lower shape (higher skewness) than low load condition. Insets show the linear fitting of the log-log scatter (see Table 1 for slope and intercept values). **(C)** Pairwise Kolmogorov-Smirnoff test (KST) for empirically estimated distributions (1–3 are Low vs. High; Low vs. Control; and High vs. Control respectively). Comparisons 4, 5 refer to the KST for each empirically estimated distribution vs. the theoretical normal for low and high cognitive load respectively. **(D)** Similar plots as in **(A–C)** in reference to the basic pointing and pointing during time estimation. **(E)** Note the different location of the scatter on the Gamma parameter plane and the difference in slope and intercept of the inset. **(F)** Pairwise comparison of empirically estimated distributions using KST for each participant: (1) baseline point vs. time estimation; (2) baseline pointing vs. normal distribution; (3) Time estimation vs. normal distribution.

The estimated Gamma parameters characterizing the PDFs of the IBI showed a distinct trend in the separation between the two conditions at 95% CI. Figure 5A shows the individualized profiling of each participant’s stochastic transitions from low to high cognitive load condition, with the arrow marking the order of those conditions. As the cognitive load increases, there is a trend across participants to increase the PDF skewness (note, the PDF shape is symmetric when the skewness value is 0), and an overall tendency to increase the variance.

The increase in the IBI’s timing variance as the cognitive load increases is reflected in the increase of the NSR (i.e., the value of the Gamma scale parameter in Figure 5B bottom panel showing the Gamma parameter shape-scale plane) across all participants. Each participant’s PDF for low (blue) and high (red) cognitive load is plotted in the inset of Figure 5A. Each point on the Gamma parameter plane represents a single participant, and the CIs are set to 95% level. Figure 5C illustrates the results of using the KST to compare two empirically estimated PDFs estimated under each condition. In particular, rows 1–3 are Low vs. High; Low vs. Control baseline pointing; and High vs. Control respectively; while the fourth and fifth rows of the color matrix in Figure 5C show the departure of the estimated PDF from the normal distribution for the low and high load condition respectively. In all cases, a significant shift of the PDF can be appreciated for different conditions performed during the same pointing task.

In contrast to the task requiring different cognitive demands, the time estimation task elicited modest changes when compared to the baseline pointing task in the estimated Gamma PDF parameters of the IBI, as is shown in Figures 5D–F. Yet, the comparison of the empirically estimated PDFs to those of the normal distribution did yield significance (Figure 5F rows 2 and 3 of the matrix). This underscores the skewed nature of these distributions and the variety of the family across the general population (see inset in Figure 5D). Although the changes in dispersion and shape were more modest in time estimation-pointing task than in the high-low cognitive load task, the overall shifts in PDF recorded within one experimental session and the variations in skewness and dispersion across subjects were quantifiable and significant.

Further distinction between the two tasks can be appreciated in the fitting line to the log-log of the scatter and the behavior of the scatter on the line. These are shown in insets to Figures 5B,E. There, the high-load case shows a broader variety of PDFs with a broader and more separable distributions per condition; while the time estimation case shows a narrower range of PDFs and a more mixed scatter of points between the baseline pointing and the pointing during time estimation. Both slopes and intercepts of the fitting line were similar (Low/High load slope −1.01 intercept −0.27; Point/Time estimation slope −1.01 intercept −0.23; also see Table 1) while the scatters shifted along the line.

**Table 1:** Power law fit of estimated gamma parameters.

Parameter	Scatter points from	Slope	Intercept
IBI	Low load vs. High load	−1.0053	−0.2703
	Point vs. Time estimation	−1.0109	−0.2278
NTM forward	Low load vs. High load	−1.0304	−0.3678
	Point vs. Time estimation	−1.0352	−0.3372
NTM backward	Low load vs. High load	−1.0178	−0.4466
	Point vs. Time estimation	−1.0409	−0.3131
AM forward	Low load vs. High load	−1.0260	−0.3856
	Point vs. Time estimation	−1.0226	−0.4055
AM backward	Low load vs. High load	−1.0003	−0.5324
	Point vs. Time estimation	−1.0133	−0.4600

### CNS Assessment of Deliberate and Spontaneous Processes in Hand Kinematics

#### Low Cognitive Load vs. High Cognitive Load

The estimated Gamma parameters characterizing the PDFs of the hand kinematics were extracted from the pointing task and separated into a deliberate forward movement segment and a spontaneous backward movement segment. Then, for each of these segments, the stochastic transitions of kinematic micro-movements were examined between the low and high cognitive load conditions.

##### Fluctuations in normalized inter-peak-time intervals (NTM)

**Figure 6:**
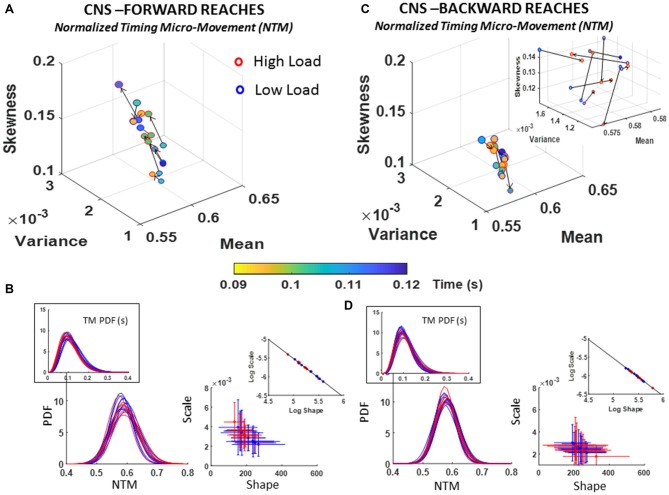
CNS voluntary control assessment of goal-directed forward normalized inter-peak-time intervals (NTM) **(A,B)** and CNS automatic backward NTM **(C,D)** during low and high cognitive load conditions. **(A)** Estimated Gamma moments showing shifts in NTM distribution parameters from low to high cognitive load condition for forward motions, and color gradient denoting the range of physical parameter (time, seconds), where marker color for each participant noticeably shifts range between conditions. **(B)** PDFs family across participants for the unitless NTM along with those for the raw TM in inset (left) and Gamma parameter plane (right). Log-log scale aligns scatter along linear fit (see Table 1 for slope and intercept information). **(C)** Same as in **(A)** for spontaneous retractions with inset plotted at local scale to appreciate the shifts in PDFs moments. **(D)** Estimated NTM PDF family along with raw TM PDFs in inset (left) and Gamma parameters (right). Inset shows the shift of the scatter along the line fitted to the log-log plot (see Table 1 for slope and intercept values and Supplementary Figure S5 for detailed comparisons of pairwise KST distribution comparisons for each participant).

The changes in the estimated Gamma moments referring to the fluctuations in timing information are shown in Figure 6. Both the deliberate (forward) and the spontaneous (retraction) motions showed a large departure from 0-shift across all participants. This means that the PDF of each participant shifted to a different PDF altogether; thus, strongly advising against the assumption of a theoretical uniform statistical approach to assess the entire group. This is the *one size fits all* model currently in use by traditional approaches and discussed in Supplementary Figure S1.

The PDFs that we empirically estimated for each participant were skewed, as is shown in the insets of Figures 6B,D; thus, strongly advising against the theoretical assumption of symmetric distributions such as the Gaussian distribution for statistical inference. Besides visual inspection, this result was further verified using the Kolmogorov Smirnov test to compare the empirically estimated distribution against the normal distribution, yielding significant departure from normality (*p* ≪ 0.01) across all participants (see Supplementary Figure S5D).

Furthermore, comparisons of the parameters of the estimated Gamma PDFs and moments yielded differences in skewness and dispersion with the task. In the forward case of Figure 6A, a trend denoting an increase in skewness and dispersion of the fluctuations in timing with the increase in cognitive demands was quantified. The large shifts in PDFs for forward motions contrasted with the backwards reach (retracting the hand to rest without instructions), where the changes were more modest (see Figure 6C). Specifically, each participant had a unique type of shift in PDF as the cognitive load increased during backward reaches (i.e., spontaneous process). Inset in Figure 6C zooms in the scatter to show the shifts in the moments of the PDF estimated and shown in Figure 6D for the backwards case.

The results of assessing these stochastic transitions with the Kolmogorov Smirnov test are detailed for each participant in Supplementary Figure S5A. Importantly, besides the shifts in PDFs, we also quantified shifts in the physical range of the parameters underlying the NMT (i.e., the range concerning the number of seconds the original inter-peak time intervals manifested). These are appreciated in Figures 6A,C, where the changes of marker-face colors should be examined following the arrow representing the order of presentation (from low to high cognitive load). The shifts in physical range correspond to the color gradient in the color bar.

To further quantify the shifts in PDFs between forward and backward cases, we used the line fitting the scatter represented in log-log transform of the Gamma parameter plane. These are depicted in the insets of Figure 6B-right panel and Figure 6D-right panel along the shape and scale axes. Forward slope −1.03 intercept −0.37; Backward slope −1.02 intercept −0.45; also see Table 1 reflect similarity in the fitting lines with different locations and spread of the scatters. In the forward motions the stochastic signatures of the NTM have a broader and more uniform spread along the line while in the backward motions the spread tended to lower NSR ranges and more symmetric shapes (down and to the right of the line). This suggests a stochastic process in the spontaneous retractions that is more predictable (towards the Gaussian ranges) and less random (away from Exponential ranges) than those quantified in the deliberate case.

##### Fluctuations in angular acceleration amplitude micro-movements (AM)

**Figure 7:**
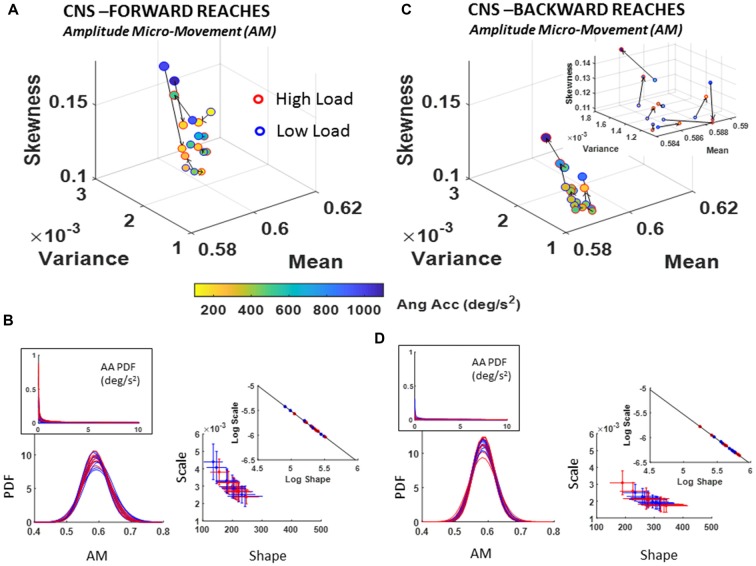
CNS voluntary control assessment of goal-directed forward AM **(A,B)** and CNS automatic backward AM **(C,D)** during low and high cognitive load conditions. **(A)** Estimated Gamma moments showing shifts in AM PDF from low to high cognitive load condition for forward motions, and **(B)** its AM estimated family of PDFs along with raw angular acceleration peaks PDF in the inset (left) and Gamma parameters (right). **(C)** Same as in **(A)** for the spontaneous retractions. **(D)** Same as **(B)**, showing the shifts in PDFs towards lower dispersion and more symmetric shapes on the Gamma parameter plane and the log-log inset showing the linear fit (see Table 1 for slope and intercept reports and Supplementary Figure S5 for detailed comparisons of pairwise KST distribution comparisons for each participant).

The analyses of the fluctuations in the amplitude of the angular acceleration, as normalized by the micro-movements data type (AM) using equation (1) show departure from 0-change in PDF for all participants. As with the TM and NTM cases, these results also advise against the use of a *grand average* treatment to these biophysical data. Each participant manifested a different stochastic shift. Further, there were no visible patterns across all participants. Here, both the forward and backwards cases showed unique shifting patterns for each person’s stochastic signatures. The trend was rather in the physical ranges of angular acceleration, which tended to decrease with the increase in cognitive load for the forward reaches. This trend in reduction of angular acceleration amplitude (Figure 7A) was generally opposite in the backwards retractions (Figure 7C), with some variations unique to each participant. The color bar (deg/s^2^) of Figures 7A,C provide information on the median physical range.

Of note is the skewed (Exponential-fit) distributions of the original peaks of the angular acceleration shown in Figures 7B,D insets. The scaling of equation (1) transformed the micro-movements data from Exponential to Gamma distributed angular acceleration micro-movements, as shown on the Gamma parameter plane and corresponding PDFs of Figures 7B,D. This family of PDFs estimated within the time span of a section further confirms that the motor signatures under cognitive load are non-stationary. They shift stochastic signatures in quantifiable ways even within the experimental session, in the first 10–20 min.

As with the NTM parameter, here, besides uncovering individual shifts between high and low cognitive loads for each movement type, it is also possible to ascertain the overall shifts of the group scatter between the deliberate and spontaneous movements. In the log-log Gamma parameter planes of Figure 7B vs. Figure 7D we can see these shifts in the slope and intercepts of the line fitting the log-log transform of the scatter (Forward slope −1.03 intercept −0.39; backward slope −1.00 intercept −0.53; see also Table 1). For the forward case there is broader spread (as in the case of NTM) than the backwards case. The latter shows a trend to PDFs with lower NSR (lower dispersion) and more symmetric shape. See also the PDF plots corresponding to Figure 7B (flatter) and Figure 7D (peakier). These features are reflected as well in lower skewness and higher kurtosis when comparing the Gamma moments across forward and backward reaches (Figure 7A vs. Figure 7C).

#### Pointing vs. Time Estimation (Decision-Making) Task

Deliberate forward and spontaneous backward reaches revealed systematic shifts in PDFs for all participants and parameters under examination, when comparing the basic pointing task to the pointing task under time estimation. Several features for each parameter are reported below.

##### Fluctuations in normalized inter-peak-time intervals (NTM)

**Figure 8:**
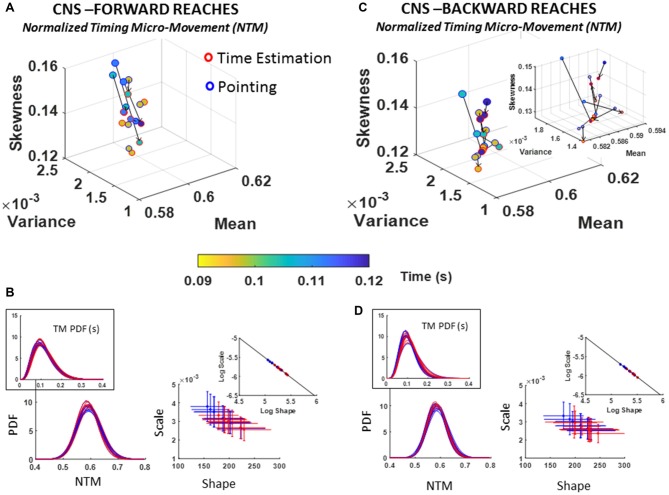
CNS voluntary control assessment of goal-directed forward NTM **(A,B)** and CNS automatic backward NTM **(C,D)** during pointing and time estimation tasks. Similar layout as in previous Figure 6.

A trend across all participants in the deliberate forward reaches was a marked decrease in parameter range (time in seconds) underlying the NTM data. Namely, basic pointing on average took longer time between peaks (blue range of the color gradient in Figure 8A) than pointing under time estimation, exhibited by shifts in color gradient towards green/yellow range. These shorter time intervals between peaks denote faster transitions in the acceleration of the hand’s rotations. Another trend with the time estimation task was a decrease in the dispersion (shown along the scale axis of the Gamma parameter plane) with most participants shifting to lower NSR and towards distributions of higher shape value (more symmetric), as shown in Figure 8B. For backward motions (Figures 8C,D), the patterns of the underlying physical time reverted, whereby marker colors in Figure 8C shifted from light to dark blue ranges for some, indicating an increase in the number of time between peaks (i.e., slower rates of rotation).

The stochastic transitions in PDF signatures were tested against the normal distribution for each estimated PDFs across conditions for both forward and backward motions and showed to significantly depart from the normality (details can be found in Supplementary Figure S5E). We obtained the slope and intercept of the line fitting the log-log transform of the scatters (Forward slope −1.04 intercept −0.34; backward slope −1.04 intercept −0.31; also see Table 1) and found a more modest shift down and to the right of the line than in the conditions involving low vs. high loads. Nonetheless, the changes can be best appreciated in the insets showing the PDFs whereby the more skewed distributions in Figure 8B as compared to Figure 8D inset are evident. Further the reduction in dispersion from inset PDFs in Figure 8B as compared to inset PDFs in Figure 8D is also evident. These visible effects were quantified and their significance shown in Supplementary Figure S5.

##### Fluctuations in angular acceleration amplitude (AM)

**Figure 9:**
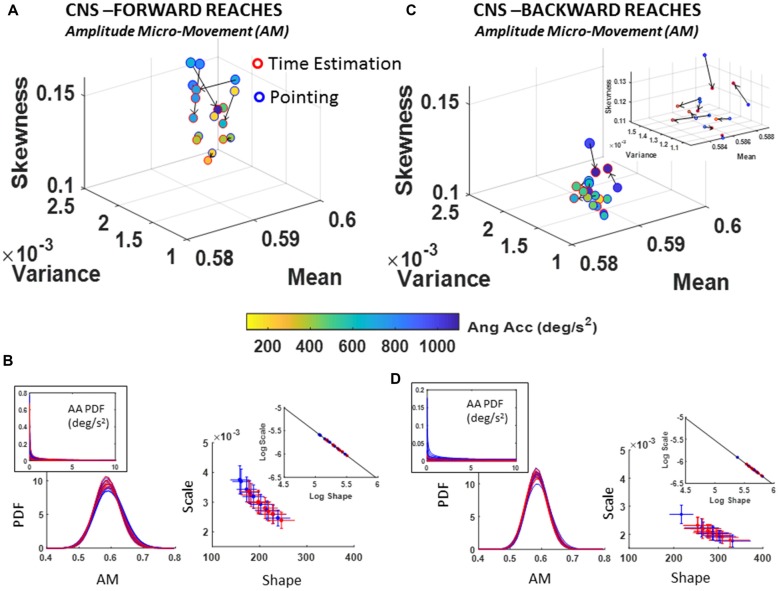
CNS voluntary control assessment of goal-directed forward AM **(A,B)** and CNS spontaneous backward AM **(C,D)** during baseline pointing and pointing during time estimation tasks. Format as in Figure 7. Note the shift of the scatter in the insets of **(B,D)** whereby the PDFs denote distributions with lower dispersion and more symmetric shapes in the backwards reaches.

A change in the estimated PDF from basic pointing to pointing during time estimation was registered for the fluctuations in the amplitude of the angular acceleration peaks, AM, as scaled by equation (1). The individual shifts in the empirically estimated Gamma moments were registered for both the forward and backward reaches. The overall trend in the scatter of forward reaches of Figure 9A is an increase in skewness of AM distributions corresponding with higher ranges of physical angular accelerations (see color gradient depicting median range values of deg/s^2^).

Comparing between the movement classes, forward reaches tended to have distributions with higher skewness as quantified in the moments graphs of Figure 9A vs. Figure 9C. Further, Figure 9B vs. Figure 9D show the differences between these deliberate and spontaneous processes reflected in the NSR (Gamma scale) and the Gamma shape estimated values for each movement class.

In the forward goal-directed motion, the stochastic signatures had higher NSR and lower shape parameters than the spontaneous backward motions. The lower shape corresponds to the higher positive skewness of Gamma moments plot in Figure 9A. These features are visualized and apparent in the corresponding panels of PDFs in Figure 9B vs. Figure 9D. They are also quantifiable as shifts of the overall scatter along the line fitting the log-log transform of the Gamma parameter plane. The slope and intercepts of the log-log transform of the scatters for the forward and backward reaches are similar (Forward slope −1.02 intercept −0.41; backward slope −1.01 intercept −0.46; also see Table 1); yet the scatter in the backwards motions shifted down and to the right towards more distributions with lower dispersion (downward-shift) and higher shape (right-shift). Furthermore, Supplementary Figure S5B points the reader to differences in the PDF between the two tasks as quantified by the KST. These differences were significant as shown in various parameters.

## Discussion

This work provides a new theoretical research framework, datatypes, and analytics combined with an experimental paradigm to study the interactions between mental states and physical actions. We systematically probed the variability inherently present in the biophysical rhythms across the multiple layers of the nervous systems, as participants pointed to communicate their decisions, and as they were exposed to different levels of cognitive loads. Under such conditions, and through a simple pointing task, we examined the influences of cognitive demands across multiple layers of the nervous systems and through fundamentally different processes—*deliberate, spontaneous and inevitable*. These proposed processes have specific characteristics and can be studied (non-invasively) through the variability of various somatic-sensory-motor and heart signals harnessed in tandem (Ryu and Torres, 2017).

We detected the effects of cognitive load in multi-modal signals across different levels of functionality, and identified specific parameters characterizing cognitive load through the stochastic shifts of biophysical signals. Specifically, using a personalized method of statistical analyses, we found families of skewed probability distributions better describing the empirical variability of the data, as opposed to assuming the normal distribution for statistical inference.

Within the time span of minutes, the stochastic signatures of parameters from the pointing motions shifted for each participant in ways that were well-characterized by a Gamma process. Using the new datatype that converts continuous analog kinematic signals to real-valued spike trains normalized between 0–1 and treating the various types of cognitive demands as a continuous stochastic process, we were able to capture marked effects of cognitive load on the somatic-motor parameters. A simple pointing task performed with the same biomechanical structure, but while making decisions on time estimations, or while counting backwards, was sufficient to help us read out in the motor and heart variability code, the various mental influences across deliberate, spontaneous and (inevitable) autonomic processes.

Of relevance here, we highlight the marked differences we found in IBI with changes in the cognitive demands of the task, transitioning from low to high cognitive loads. Changes in PDF of the IBI were not as marked with the time estimation task, perhaps inviting thoughts about differences between a task with higher cognitive demands (counting backward) and one with lower demands (estimating time). Indeed, the mere quantification of the number of angular acceleration peaks during the higher cognitive loads denoted higher demands in bodily motions: i.e., the hand moved significantly more at the micro-level with higher cognitive demands. As such, when viewed cumulatively over the timespan of the task, the system overall required more energy. This may be reflected as well in the shifts in IBI with higher cognitive loads. In this sense, we found a statistically quantifiable link between cognitive loads and physical motions whereby differences are detectable and have characteristic values that we summarized in various ways. Among these, the slope and intercept of the log-transform of the scatter points on the Gamma plane was similar for each experiment (i.e., low-high cognitive load vs. pointing and pointing while estimating time), denoting a power-law relation between the shape and scale estimated parameters for each participant. Yet the location of the scatter along the fitting line of these points representing the personalized family of PDFs changed across conditions and between the deliberate and spontaneous classes of motions. They also changed for the IBI timings of the autonomic motions. The main shift from deliberate to spontaneous mode was down and to the right on the Gamma parameter plane, consistently denoting distributions with lower NSR and more symmetric shapes. During these tasks, across all parameters, the spontaneous retractions were unexpectedly more controlled (lower noise and higher predictability) than the forward ones. Yet it was the forward motions that broadcasted more clearly the shifts across conditions in the signatures of variability of the kinematic parameters. These shifts were also noted in the IBI activity.

One limitation of the present methods is that they depend on the sampling resolution of the sensors and accordingly, on the time length of the task. In this study, due to time constraint of the experiment (to avoid fatigue) we were only able to examine angular acceleration as a kinematic parameter. As explained in the methods and Supplementary Figure S2, within the time constraints of this task, the angular acceleration provided enough peaks in its waveform for statistical power in our distribution-parameter estimation (we needed above 100 peaks for tight CIs). If we were to conduct this experiment for a longer period, we could examine other position-related parameters, such as linear speed and hand trajectory curvatures. However, this would have caused participants to experience fatigue during a prolonged experiment with conditions involving multiple levels of cognitive loads. The linear speed has fewer peaks-though we have recently studied the micro-movements in the context of basic pointing in autism (Wu et al., 2018). To make these methods amenable to use with commercially available biosensors like the inertial measurement units (IMUs embedded in smart phones) we could use linear acceleration. Linear accelerations would provide us with sufficient number of peaks in its waveforms, so we could possibly use these wearable devices in future studies. The trade-off is that we would then lose the positional data that we have access to with the present research-grade sensors.

The main take home message from the study is that even subtle fluctuations in timing and amplitude of the biophysical signals that we recorded could be detected under the proposed framework. Thus, the exact same task funneled out very different stochastic scenarios under slightly different conditions. The mere act of having to decide or having to do so under different cognitive loads changed these heart and kinematics parameters in ways we could capture here. These stochastic shifts would have been missed if we had averaged across the group or relied only on observation. In this sense, the present methods allowed us to detect *change* at the individual level on more than one statistical dimension. It also allowed us to examine the cohort as a group and within each condition, look at the effects of the cognitive demands on the deliberate, spontaneous and autonomous functional somatic-motor classes.

We underscore here that these stochastic shifts in the biophysical parameters were empirically characterized. We did not assume* a priori* any PDF, nor did we assume stationarity of the random processes under examination (Supplementary Figure S1). The shifts in the *empirically estimated* parameters of the continuous Gamma family of probability distributions, that we quantified here, occurred on the time scale of minutes, i.e., the duration of the experimental session. They strongly suggest that prior assumptions involving kinematics data analyses may be insufficient to capture the richness of cognitive phenomena, pertaining to their effects on the somatic-motor signals. Here, we showed that cognitive phenomena do not merely elicit a change in the mean or variance of somatic-motor related variables under a single PDF. Rather, different PDFs are needed altogether to better characterize cognitive phenomena for each person under examination, as it is continuously funneled through physical activity that leads to shifts in the signatures. The process changes dynamically along the multiple layers of the nervous systems.

Another aspect of the results alludes to the motor control literature examining pointing behavior. There, the uninstructed retraction segments, during which the hand automatically returns to rest (i.e., backward movement), are hardly ever considered as part of the overall behavior. However, when we examined those retraction segments, we quantified the effect of cognitive load on biophysical signals from spontaneous processes in the moment-by-moment variations of the angular acceleration NTM. Indeed, their stochastic signatures showed statistically significant shifts in the empirically estimated parameters of the Gamma PDF family (i.e., statistically significant departure from zero-valued change across all participants for all Gamma moments).

Pointing is a very automatic task, and yet several motor signals from the peripheral end-effector were significantly affected by simply adding an additional task requiring decision-making (see Supplementary Figure S5 whereby for each participant at least one change in PDF is highly significant across pairwise compared conditions). This result implies that decision-making processes driven by central controllers at the CNS level can be quantified using the continuous flow of the motor signal, as the voluntarily generated bodily motions unfold to communicate the decision; and as the fast-automatic segments of the motion spontaneously unfold. In this sense, a parallel between slow-fast (deliberate-automatic; Kahneman, 2011) decision-making processes and deliberate-spontaneous somatic-motor signals can be established and well characterized using continuous physiological signals beyond discrete mouse clicks. Our conceptualization of multi-layered influences across the different functional levels of the nervous systems adds the *inevitable* (autonomic) afferent processes feeding back to the cognitive systems. As such, we provide a new experimental paradigm and a unifying statistical framework to study *embodied cognitive decision-making* under a renewed theoretical construct of multi-modal, multi-functional *recursive* kinesthetic afference.

## Embodied Approach to Study Cognition

The current study employs a novel methodology to assess features of* embodied* cognition. The new method extracts continuous signals obtained from the PNS, including the ANS, and statistically characterizes those signals under a common unit-less (i.e., normalized) scale, using different levels of cognitive loads (driven by the CNS), thereby allowing us to gain a glimpse into the *brain-body coupled stochastic dynamics*. In this sense, we have characterized cognitive load with sensory and somatic-motor signals, alluding to processes that occur in a closed (recursive) loop between the many layers of the brain and the body (the body that the brain aims to control at will); including also those spontaneous processes that fall largely beneath observational and/or sensing awareness. For instance, the input signals from the micro-movements of the movement-kinematics and the heart signals can be thought of as fluctuations providing an important source of guidance to the brain. They may be a form of re-afferent feedback, to help the brain compensate for synaptic transductions and transmission delays. By selectively shifting the signatures of statistical variability under different levels of cognitive load, different functional relations (in terms of probabilistic maps) between bodily responses and environmental demands (including cognitive loads) may be built, to be able to predict ahead the sensory consequences of bodily actions, even in the absence of, or the intermittent availability of relevant sensory information.

This multi-layered, multi-modal and multi-functional embodied approach to the study of cognitive processes has the potential to provide a more holistic perspective on our overall understanding of cognition and its development. Indeed, this simple paradigm was useful to examine the changes in bodily signals across multiple layers of the nervous systems and characterize the sensory-motor behavior that underlies cognitively driven performance. Furthermore, by adopting the renovated kinesthetic reafferent framework in this study, we could capture the variations of motor and multifaceted sensory inputs that must be integrated to drive cognitive processes (e.g., goal-selection, planning, decision making) under varying levels of control, ranging from voluntary to automatic to autonomic.

## Conclusion

The current study provides important evidence to justify an embodied and personalized approach to studying cognition (Gallagher, 2014). This study offers a renovated theoretical construct grounded on the principle of kinesthetic reafference, a new unifying statistical method, datatypes and experimental paradigms to assess voluntary, automatic and autonomic signals through a common lens. As such, this work is an invitation to use such tools to help advance the field of embodied cognition.

## Author Contributions

JR designed experiment, collected and analyzed data and wrote the article. EBT designed experiment, designed analyses and wrote the article. Both authors agreed to the final version of the manuscript.

## Conflict of Interest Statement

The authors declare that the research was conducted in the absence of any commercial or financial relationships that could be construed as a potential conflict of interest.
