# Supplementary material for: Characterization of Sensory-Motor Behavior Under Cognitive Load Using a New Statistical Platform for Studies of Embodied Cognition
Source: Front Hum Neurosci. 2018 Apr 6;12:116. doi: 10.3389/fnhum.2018.00116 (PMC5897674; doi:10.3389/fnhum.2018.00116)
Supplement: Supplementary file 1 [file Data_Sheet_1.docx]

Characterization of sensory-motor behavior under cognitive load using a new statistical platform for studies of embodied cognition

Jihye Ryu^1^, Elizabeth B. Torres^2^

# Supplementary Material

## S1. Personalized approach vs. one-size fits all (grand average) approach

| **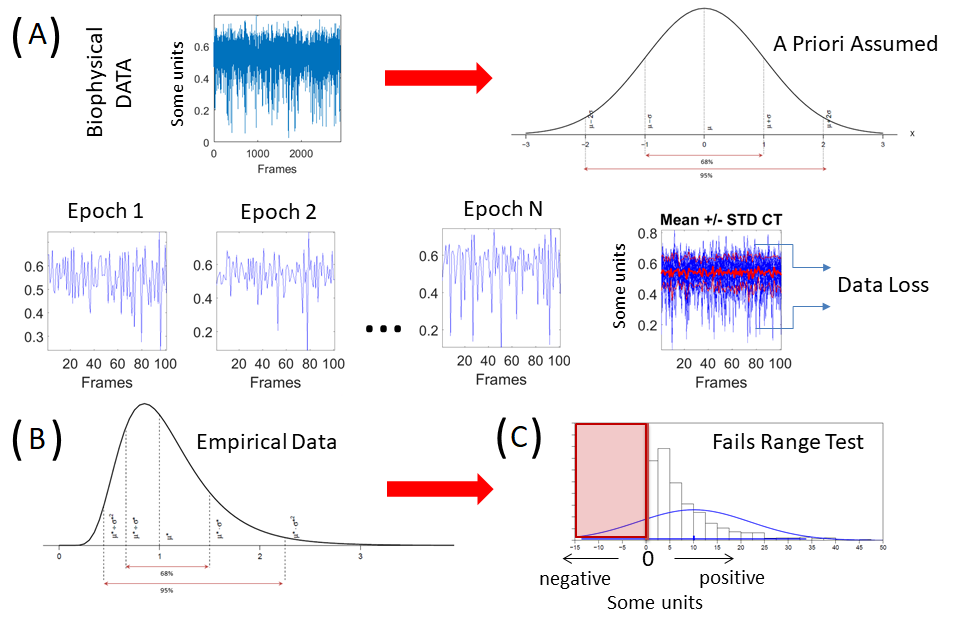** |
| --- |
| **S1. Comparison between traditional analytics and a personalized statistical approach (Statistical Platform for the Individualized Analyses of Behavior - SPIBA) used in the current study** **(A)** Raw biophysical data in the form of time series of peak and valleys of some parameter are traditionally broken down into epochs, stocked up and averaged under the assumption of normality (e.g. using the theoretical Gaussian mean.) This grand average method incurs in great data loss because it smooths out as noise the minute fluctuations in the amplitude and timing of the original data (self-generated by the nervous systems biorhythms) As such we lose the very information we are after. This is the “one size fits all” statistical model in use across the health and brain sciences. (B) The fluctuations in the data are not normally distributed. (C) The data of interest under a Gaussian assumption fail the range test, whereby the mean +/- 2 times the standard deviation may even reach spurious values, non-existent in the empirical data. |

## S2. Four types of peaks’ profiles from the same motion

**
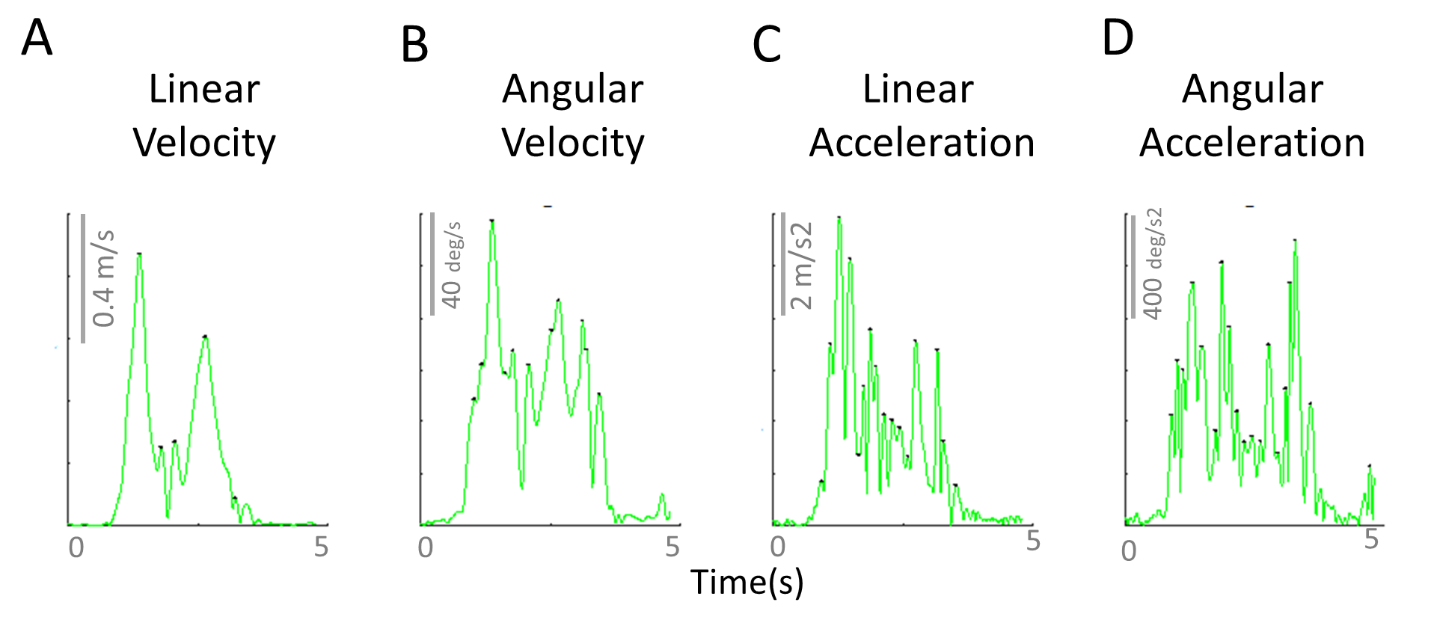
**

**Figure S2.** Four types of speed profile of a typical pointing movement. **(A)** Linear velocity **(B)** Angular velocity **(C)** Linear acceleration **(D)** Angular acceleration. Because angular acceleration showed to have the largest number of peaks during a single pointing movement, peak data obtained from the angular acceleration speed profile was analyzed, as this would provide the highest statistical power for the MLE process.

| S3. MLE for kinematic parameters **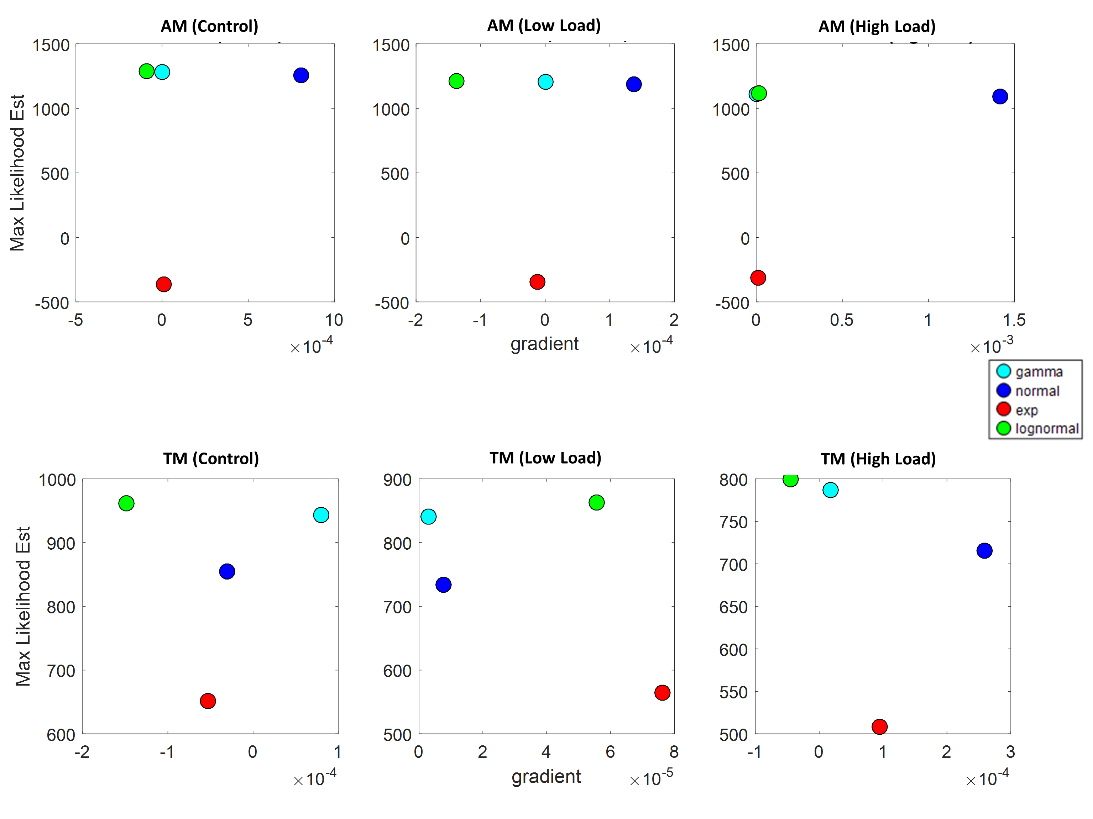** |
| --- |
| **Figure S3.** Maximum likelihood estimated values for AM (top) and TM (bottom) for 3 different conditions (in columns), for a typical participant. The horizontal axis contains the value of the gradient at the end of the optimization process (-5x10^-4^ ~ 1.5x10^-3^ range according to the set tolerance value of 10^-30^ for the optimization process). The vertical axis contains the maximum likelihood estimation value for the Gamma, normal, exponential and lognormal distributions (see legend). The respective values are: AM for control condition [1279.0,1256.6,-366.9,1288.7], AM for low load condition [1203.9,1186.6,-345.2,1211.2], AM for high load condition [1111.0,1090.4,-314.2,1120.1], TM for control condition [943.4,854.7,651.7,961.3], TM for low load condition [841.1,733.5,564.9,863.4], TM for high load condition [787.1,715.0,508.7,799.7]. |

| S4. MLE for inter-beat interval **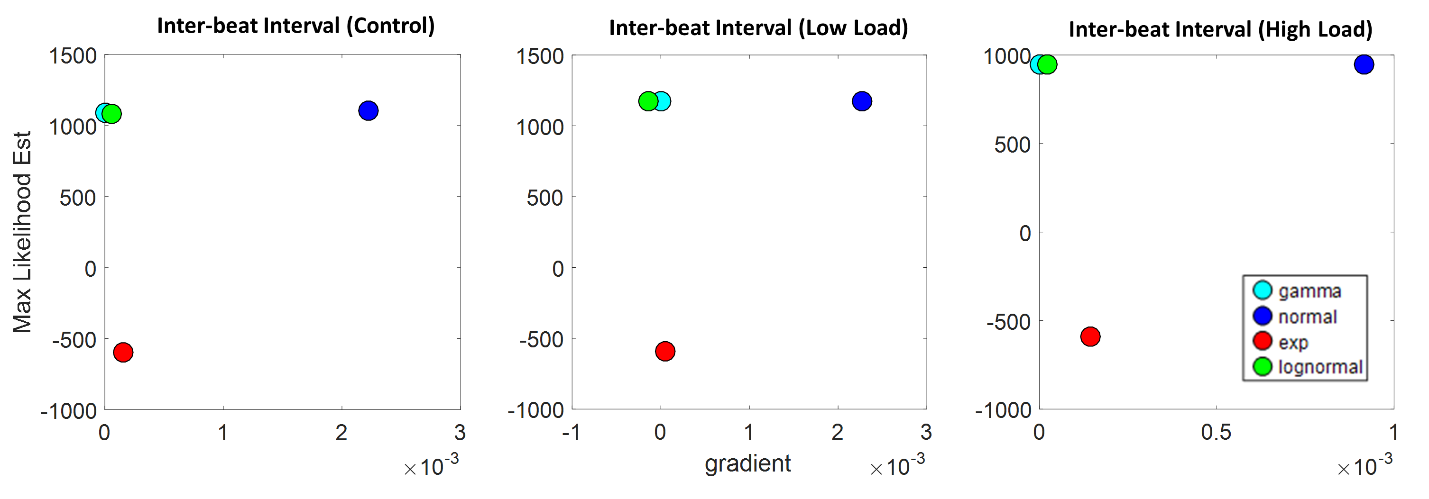** |
| --- |
| **S4.** Maximum likelihood estimated values for inter-beat interval for 3 different conditions, for a typical participant. The horizontal axis contains the value of the gradient at the end of the optimization process (-1x10^-3^ ~ 3x10^-3^ range, according to the set tolerance value of 10^-30^ for the optimization process). The vertical axis contains the maximum likelihood estimation value for the Gamma, normal, exponential and lognormal distributions (see legend). The respective values are: control condition [1090.3,1100.8,-595.1,1083.9], low load condition [1175.9,1176.1,-590.1,1175.2], high load condition [946.75, 944.99, -589.92, 946.65]. |

## S5. Kolmogorov-Smirnoff Test

**
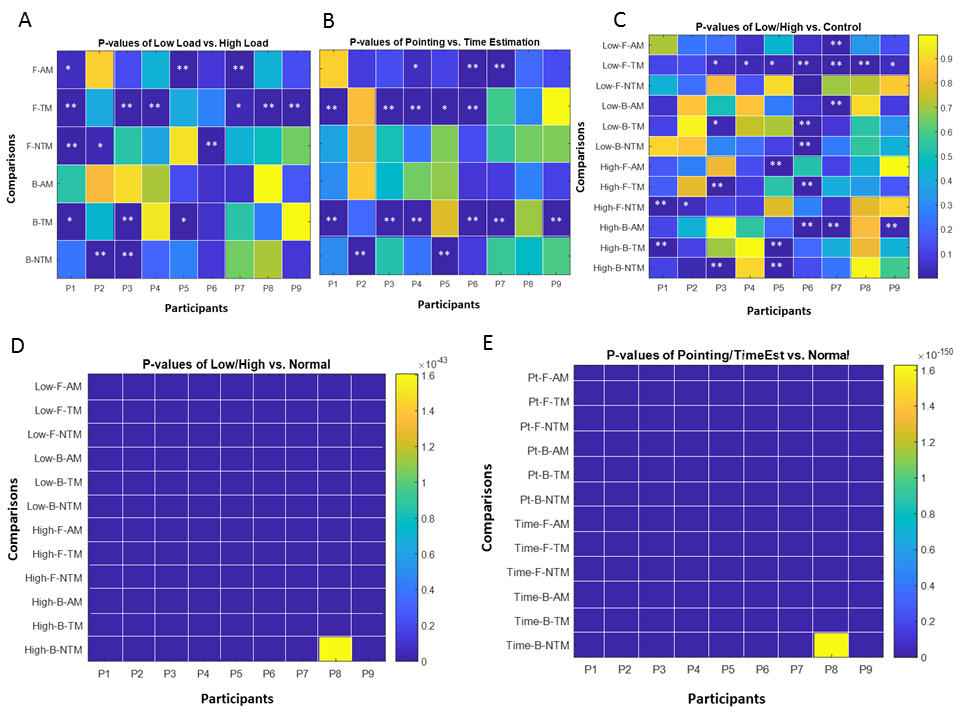
**

|  |
| --- |
| **Figure S5.** KST for empirically estimated distributions compared pairwise between conditions. (A) High vs. low cognitive load: rows are condition (forward – amplitude micro-movements, F-AM; forward time, F-TM; forward normalized micro-movements time, F-NTM; backward– amplitude micro-movements, B-AM; backward time, B-TM; backward normalized micro-movements time, B-NTM) and columns are participants P1-P9. P-values of the test are shown along the color gradient of the color bar, where * indicates p<0.05, and ** indicates p<0.01. (B) Same as in (A) for the baseline pointing and the pointing during time estimation. (C) Comparisons of low/high load condition against the control condition (first 6 rows: low load vs. control for forward AM, Low-F-AM; low load vs. control for forward TM, Low-F-TM; low load vs. control for forward NTM, Low-F-NTM; low load against control for backward AM, Low-B-AM; low load vs. control for backward TM, Low-B-TM; low load vs. control for backward NTM, Low-B-NTM; last 6 rows correspond to the high load vs. control comparison with same metrics as the low load vs. control comparison). Note that for each participant at least one entry of each matrix is significant. (D-E) are the results of the KST for each estimated distribution vs. the normal distribution. Note that all entries are close to 0 indicating non-normally distributed data for amplitude and timing. |

**Table S1.** Median time (ms) elapsed to complete a pointing gesture (composed of a forward and backward movement segment) for each condition

| Participant | Low Load | High Load | Pointing | Time-Estimation |
| --- | --- | --- | --- | --- |
| P1 | 1325 | 1535 | 1415 | 1405 |
| P2 | 1570 | 1620 | 1675 | 1765 |
| P3 | 1210 | 1740 | 1368 | 1340 |
| P4 | 1475 | 1655 | 1483 | 1370 |
| P5 | 1520 | 1535 | 1598 | 1510 |
| P6 | 1190 | 1395 | 1445 | 1333 |
| P7 | 1845 | 1943 | 1865 | 1985 |
| P8 | 1405 | 1760 | 1750 | 1565 |
| P9 | 1763 | 2145 | 1820 | 1770 |

**Table S2.** Pairwise comparison test results of number of peaks in angular acceleration

| Comparison | Motor Segment | t-value | p-value |
| --- | --- | --- | --- |
| Low Load vs. High Load | Forward Low vs. Forward High | T(8) = 4.37 | <0.01** |
|  | Forward High vs. Backward Low | T(8) = 4.42 | <0.01** |
|  | Backward Low vs. Backward High | T(8) = 4.22 | <0.01** |
|  | Forward Low vs. Backward Low | T(8) = 1.33 | 0.26 |
|  | Forward Low vs. Backward High | T(8) = 4.17 | <0.01** |
|  | Forward High vs. Backward High | T(8) = 2.00 | 0.09 |
| Pointing (Pt) vs. Time Estimation (Time) | Forward Pt vs. Forward Time | T(8) = 4.59 | <0.01** |
|  | Forward Time vs. Backward Pt | T(8) = 1.00 | 0.35 |
|  | Backward Pt vs. Backward Time | T(8)= 2.77 | 0.03* |
|  | Forward Pt vs. Backward Pt | T(8) = 0.14 | 0.89 |
|  | Forward Pt vs. Backward Time | T(8) = 0.72 | 0.49 |
|  | Forward Time vs. Backward Time | T(8) = 0.24 | 0.81 |

***p<0.01; *p<0.05*

**Table S3.** Pairwise comparison test results of time from being prompted to reaching the target

| Comparison | t-value | p-value |
| --- | --- | --- |
| Control vs. Low Load | T(8) = 0.84 | 0.43 |
| Low Load vs. High Load | T(8) = 0.15 | <0.01 ** |
| High Load vs. Control | T(8) = 3.53 | <0.01 ** |

***p<0.01; *p<0.05*

**Table S4.** Pairwise comparison test results of error in time estimation

| Comparison | t-value | p-value |
| --- | --- | --- |
| Control vs. Low Load | T(8) = 0.18 | 0.86 |
| Low Load vs. High Load | T(8) = 4.21 | <0.01 ** |
| High Load vs. Control | T(8) = 2.89 | 0.04 * |

***p<0.01; *p<0.05*
